# Warthin-like variant of papillary thyroid carcinoma with lymph node metastases: a case report and review of the literature

**DOI:** 10.1186/s13256-023-04313-1

**Published:** 2024-01-15

**Authors:** Andrii Hryshchyshyn, Andrii Bahrii, Pavlina Botsun, Volodymyr Chuba

**Affiliations:** 1 Medical Center Hormony, Vinnytsia, Ukraine; 2 Laboratory of Pathology “CSD Health Care”, Kyiv, Ukraine

**Keywords:** Warthin-like variant, Thyroid carcinoma, Papillary thyroid carcinoma, Warthin-like papillary thyroid carcinoma, Case report, Thyroid surgery

## Abstract

**Background:**

We present a rare case of thyroid lesion marked as the Warthin-like variant of papillary thyroid carcinoma (WLV-PTC) with lymph node metastases. A proper preoperative identification is difficult because of unspecific cytology features and common ultrasound characteristics of this malignant tumor. The long-term prognosis cannot be thoroughly described due to the scarcity of data. The purpose of the presentation is to show common characteristics and long-term survival rates of an uncommon variant of differentiated thyroid cancer (DTC). Therefore, the data represented in this article can make a significant contribution to future investigations.

**Case presentation:**

A 40-year-old Ukrainian woman had a lesion in the thyroid gland, which was accidentally diagnosed during medical checkup. Ultrasound (US) features were similar to the common suspicious nodule. It had typical signs of suspicion for malignancy (TI-RADS-4) on the background of thyroiditis. A thorough investigation of the neck showed lymph nodes with nonspecific US features on both lateral compartments. Lymph nodes were hypoechoic, oval-shaped and 10 mm wide, with regular contours, low central vascularity, with preserving hilar fat, without cystic formation. The patient did not have any complaints or changes in the hormone status. No hereditary findings linked with cancer were discovered. The woman had been living for a long time in the country with a high level of insolation, which was atypical for the ordinary environment of the patient. Fine-needle aspiration (FNA) of the lesion was done and the Bethesda system 6 result was obtained. Total thyroidectomy with central lymph node dissection was accomplished. The histological conclusion was WLV-PTC on the background of lymphocytic infiltration of the gland with metastasis to the lymph nodes. The inpatient radioactive iodine (RAI) ablation (100 mCi) was subsequently performed. Hormone withdrawal was used followed by RAI. In one year after the surgery the level of thyroglobulin (Tg) was 0.2 ng/ml. Up to the present time the five-year follow-up has not demonstrated any signs of recurrence relying on a level of Tg (< 0.04 ng/ml), Tg antibodies (< 14 IU/ml), neck US without any structural disease.

**Conclusion:**

WLV-PTC resembles salivary gland tumors with similar histological features. This variant is not well known, but often associated with a stroma lymphocytic infiltration and a low risk of lymph node metastases. It is regarded that this rare subtype has similar long-term survival rates as classic papillary thyroid cancer (PTC).

## Introduction

Differentiated thyroid cancer (DTC) is a common type of malignancy of the thyroid gland. More than 80% of all thyroid cancer cases consist of papillary carcinoma [1]. According to the World Health Organisation (WHO) data, there are 8 subtypes of papillary thyroid cancer (PTC): infiltrative follicular, tall cell, columnar cell, hobnail, solid, diffuse sclerosing, Warthin-like, and oncocytic [2]. We represent a case of such as a unique subtype of PTC as the Warthin-like variant obtained by 40-year-old woman. In the vast majority of cases, a tumor is diagnosed in women aged 30 to 50 [3]. Kim *et al.* revealed 0.2% presence of the Warthin-like variant of papillary thyroid carcinoma (WLV-PTC) in 8179 patients [4]. Therefore, there is a lack of data regarding long-term prognosis and tumor behavior in the literature. Approximately 95 patients were described in published articles with the abovementioned pathology subtype. The presence of the papillae structures lined with malignant oncocytic cells and lymphoid stroma are the main characteristics of the WLV-PTC [5]. The first description of the WLV-PTC was made by Apel in 1995. He defined the existence of oxyphilic cells on the background of the stroma lymphocytic infiltration. This archetype resembles papillary cystadenoma lymphomatosum of the salivary gland [6]. Local lymph node metastases are relatively rare (22%) comparing to classic PTC [7].

The case report confirms common histological characteristics of the WLV-PTC, its epidemiology, influence on overall and disease-specific survival. The five-year disease-free survival was also assessed and established.

## Case presentation

A 40-year-old Ukrainian female had a check-up and the ultrasonography (US) of the neck was performed. Previously, no complaints of the presence of lumps or pain zones were recorded. The patient had lived in a highly insolated climate zone for several years. She did not report a history of radiation exposure or thyroid diseases among her relatives.

### Clinical findings

US of the neck noted hetero echogenicity of the thyroid gland with distinct areas of hypo- and hyperechogenicity. The volume of the gland was 8.3 cm^3^. Also, a small hypoechogenic 8 mm wide nodule with an irregular, spiculated margin and with microcalcifications was found in the left lobe. The nodule was taller than wide, which is a specific feature of malignancy. Also, the US of the neck detected small lymph nodes (less than 10 mm) in the bilateral compartments, which were hypoechoic, oval with a smooth margin, echogenic hilus and hilar vascularity. In the central compartment, no suspicious lymph nodes were found.

A fine-needle aspiration (FNA) biopsy was performed several days after the pathology had been found. The result belonged to Bethesda category 6 (malignant). FNA of the nodules in the lateral compartments was not performed because of absence of the suspicious signs during the US. The main laboratory tests that could affect the thyroid function were as follows: thyroid-stimulating hormone (TSH)—2.28 mU/L (reference 0.45–4.0), free thyroxine (T4)—0.7 ng/dL (reference 0.8–1.8), thyroid peroxidase antibodies (TPO Ab)—544 IU/ml (less than 9), ionized calcium—1.23 mmol/L (reference 1.16–1.31).

The patient underwent total thyroidectomy (TT) with central lymph node dissection (CLND) one month after the pathology had been detected. The Delphian’s node, pretracheal lymph nodes and fat tissue were dissected.

**Fig. 1:**
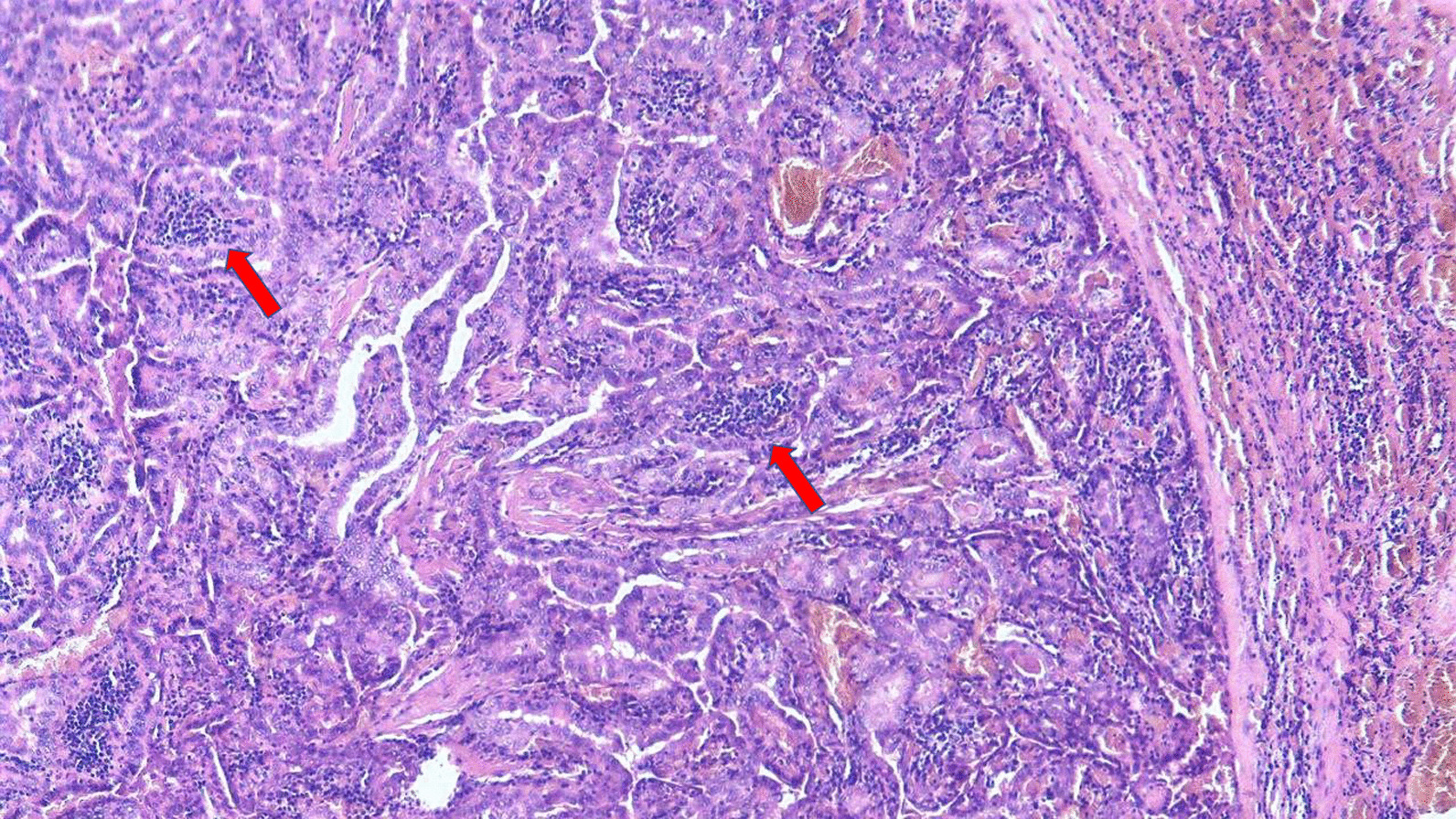
Microscopic features of the patient's WLV-PTC tumor. Lymphoplasmacytic infiltration in the papillary stalks is marked by a red arrow

**Fig. 2:**
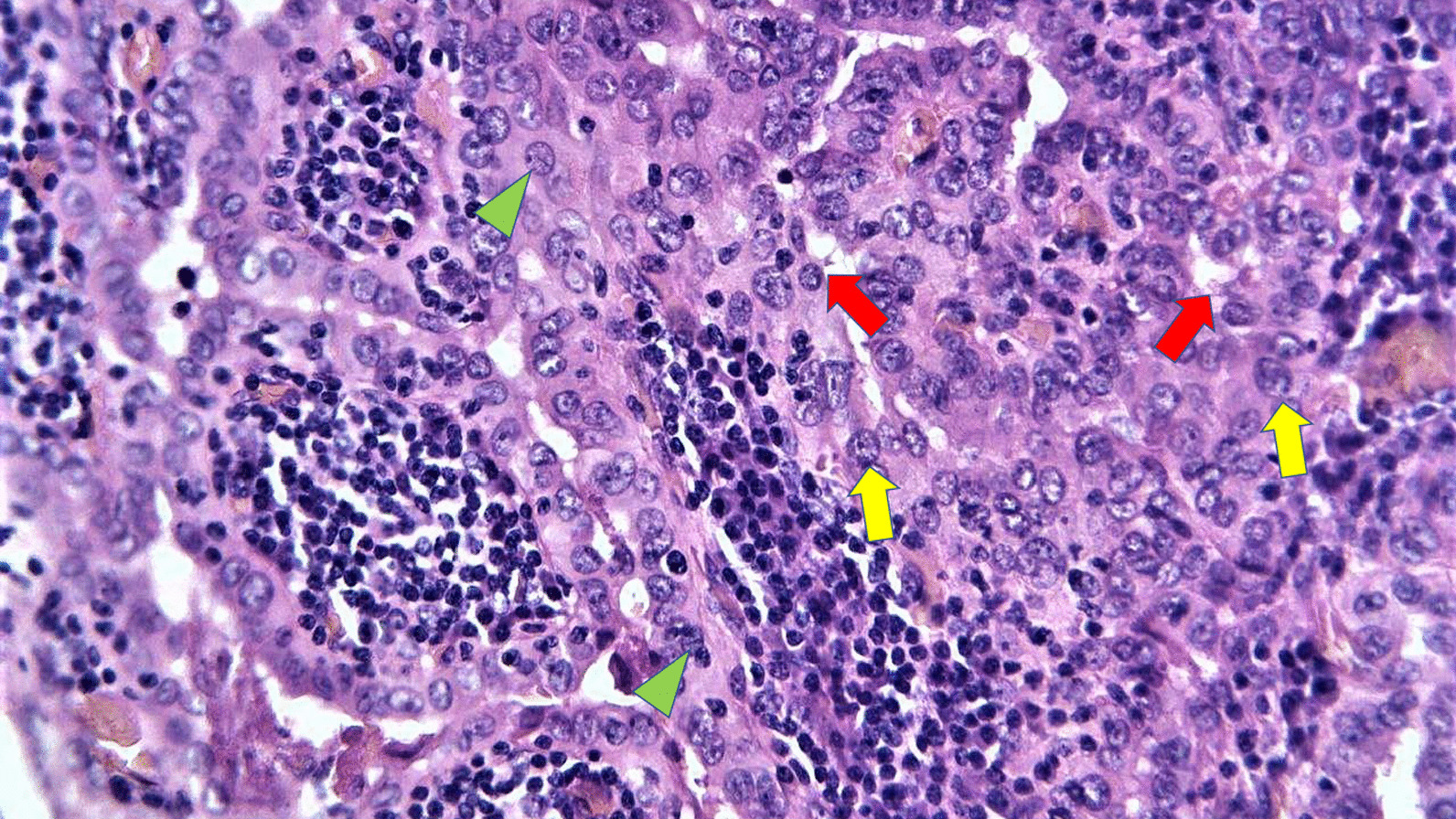
Microscopic features of the tumor's cells: polygonal oncocytes (red arrows), nuclear enlargements (yellow arrows), and grooves (green spearheads)

Histopathology revealed typical features of the Warthin-like papillary thyroid carcinoma (ICD-O 8260/3): oxyphilic follicular cells which were lined out on the papillae structure; infiltrative lymphocytic stroma; with the lymphatic invasion of the tumor (Figs. 1 and 2). Also, one of 5 dissected lymph nodes was diagnosed as metastatic. The diagnosis was confirmed as pT1aN1aM0 I stage. The patient was discharged the next day. Medications with calcium and vitamin D were prescribed for 6 months.

**Fig. 3:**
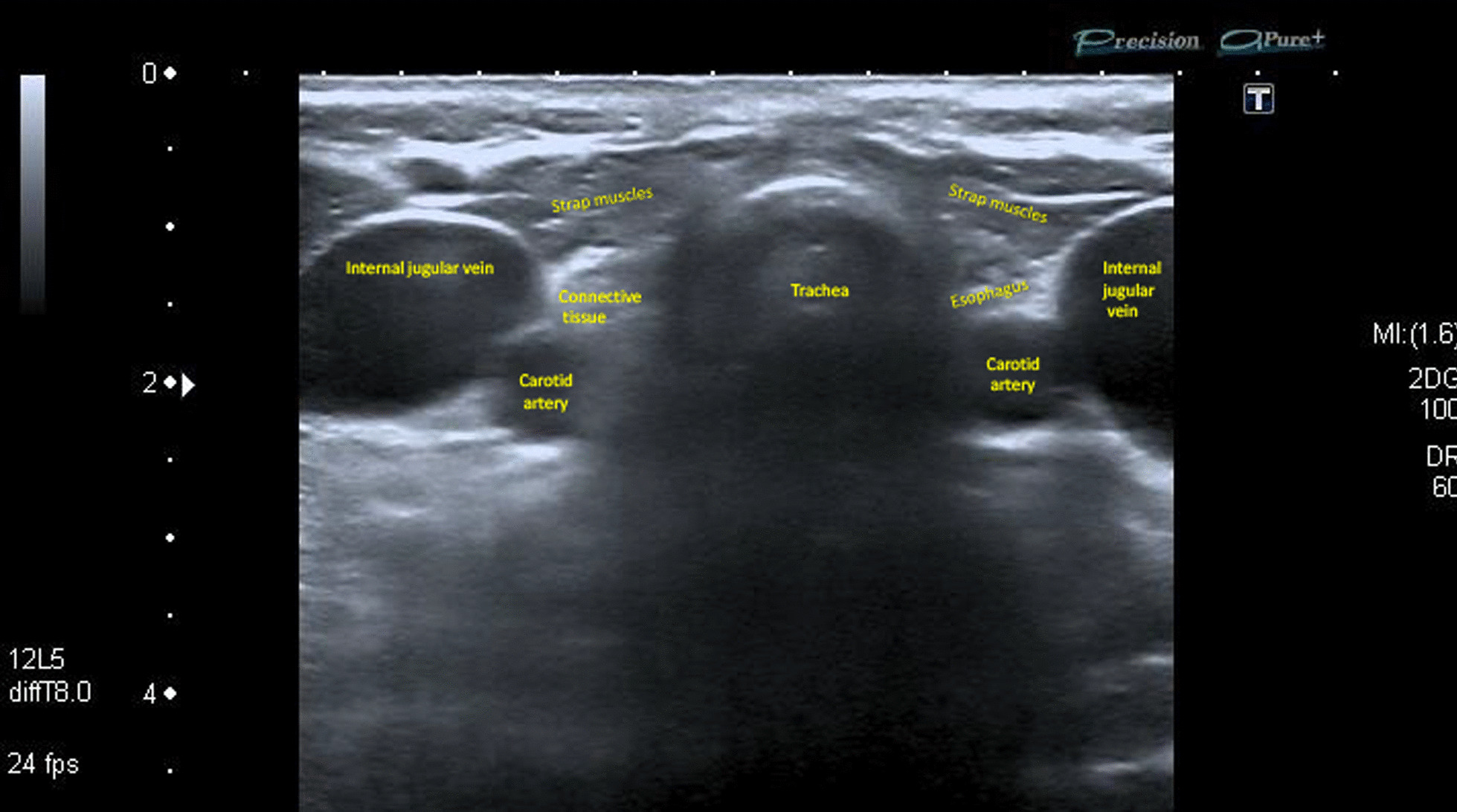
US image of the patient’s neck 5 years after total thyroidectomy with central lymph node dissection. The right side of the thyroid bed is filled with connective tissue. Additional thyroid remnants were not detected (The main structures were signed in the image)

One month after the surgery, inpatient radioactive iodine (RAI) ablation was conducted with a 100 mCi dosage. Thyroxine withdrawal was used to achieve hypothyroidism. One month after TSH equaled 75.0 mU/L. No other complications following RAI were detected. In 5 months after the surgery laboratory tests were as follows: TSH—0.11 mU/L (reference 0.45–4.0), Tg—0.2 ng/ml (disease-free reference < 0.2), Tg Ab—20 IU/ml (reference < 40). No proof of recurrence or persistence of the disease was detected on the US of the neck. L-thyroxine was prescribed to achieve a 0.5–2.5 mU/L TSH level. The five-year follow-up identified the absence of the recurrence. It was confirmed by neck US with no evidence of structural disease, a Tg test (< 0.2 ng/ml) and a Tg Ab test (<4 IU/ml). Suspicious US changes of the neck lymph nodes were also not detected (Fig. 3).

The CARE guidelines were applied in writing this report.

## Discussion

WLV-PTC is one of the 8 subtypes of classical PTC according to the WHO [2]. This tumor develops mainly in women with an average age of 50 years [7]. The first description of the cases of WLV-PTC in English literature was made by Apel *et al.* in 1995. It was noted that this type of pathology resembled papillary cyst adenoma lymphomatosum of the salivary gland [6]. As a result, we added to the literature one new case of WLV-PTC with lymph node metastases.

Clinical presentation of the pathological variant of the malignancy is the same as in classic PTC [8]. Our patient also did not have any symptoms of the disease, even with the presence of the Hashimoto thyroiditis. The nodule was identified accidentally. The high rate of TPO Ab (> 500) did not cause hypothyroidism and following complaints. Annual check-up should be conducted to avoid missing of an occult malignancy. On the other hand, an overdiagnosis may lead to performing unnecessary surgeries with following complications.

US findings indeed may differentiate a suspicious lesion from a benign one. Conducting a study, Ning *et al.* pointed out some features of WLV-PTC nodules among 32 cases. The author determined that in 97% of the cases, nodules were solid or nearly solid, and in 78.8% they were hypoechoic [9]. In our report, these assumptions were confirmed. US is considered as the main method of decision-making about applying FNA biopsy. Thyroid Imaging, Reporting and Data System Lexicon Directory (TI-RADS) can be used in routine assessment of malignant nodules. Patients with TI-RADS category 4 and 5 lesions should unequivocally be undergone biopsy because of the high level of positive predictive values [10]. However, preoperative cytological definition of WLV-PTC is highly problematic.

FNA should be performed in all suspicious thyroid lesions. This method could identify the exact genesis of the pathology. Detection of the WLV-PTC utilizing FNA may not be pointed out because of overlapping features of lymphocytic infiltration, oncocytic cells and papillary nuclear signs [11]. The cytological pattern of the WLV-PTC can imitate distinct pathologies, such as: Hurtle cell carcinoma, oncocytic variant of PTC, lymphocytic thyroiditis and follicular lesion [12]. Missaoui *et al.* examined 150 patients with WLV-PTC who made up 87.5% Bethesda categories 5 and 6 [3]. The result of FNA of the presented patient was Bethesda category 6 (malignant). Our patient also obtained malignant cytology which confirms common statistics. As mentioned above, the tumor was less than 10 mm, but nonetheless highly suspicious lesion should be biopsied. However, preoperative correct identification of the subtype of a tumor may be beneficial to choose the appropriate treatment.

Predominantly macroscopic detailing of the nodule showed a grey lesion with undefined, irregular, and sharp margins [7]. Also, this pathology had the same patterns outlined by the gland.

A method of surgery should be chosen according to the size of a tumor, FNA results, US examination of the neck, the medical history of a patient and clinical findings. In our case, we chose total thyroidectomy with CLND as we had previously done in similar cases. The choice was based on the cytology, presence of thyroiditis and opinion of the patient. Thereby, we removed metastatic nodules, but its impact on long-term survival has not been known because of rarity of the pathology.

Paliogiannis *et al.* (2012) reviewed the data of several case series. Among 54 patients with WLV-PTC, local lymph node metastases were found in 22% of cases [7]. However, Rotstein (2009) revealed that the incidence of occult nodal metastases of PTC varied between 30 and 90% [13]. The necessity to do lymph node dissection in similar cases is still controversial to date. According to mentioned rates, we have decided to perform CLND in this particular case. It possibly avoids any local recurrence or persistent disease in the future.

Probably, gene mutation may facilitate the decision-making process to determine the volume of a surgery. Absence of a cheap mutation-based gene set limited an opportunity to detect common BRAF V600E mutation. The number of articles about advanced WLV-PTC, cases with long-term disease-free survival is not enough represented in literature. A deeper analysis of tumor’s behavior will help to choose the most effective treatment option.

## Conclusion

Our case report confirms the main signs of manifestation of the WLV-PTC. The most important are as follows: indolent existence of lesions, absence of clinical symptoms, chronic lymphocytic thyroiditis as a background, and presence of specific histological patterns (papillae which are lined with oncocytic cells, nuclear grooves, and pseudoinclusions). The five-year disease-free survival seems to be favorable for that pathology. Neither performing nor avoiding CLND could be strongly recommended due to the scarcity of data about the incidence of local lymph node metastases. More information of this subtype of PTC should be collected to understand metastasizing, long-term survival rates and subsequent appropriate treatment.

## Data Availability

The author will provide all the data on request.

## Abbreviations

WLV-PTC: Warthin-like variant of papillary thyroid carcinoma
DTC: Differentiated thyroid cancer
US: Ultrasound
TI-RADS: Thyroid Imaging Reporting and Data Systems
FNA: Fine needle aspiration
RAI: Radioactive iodine
Tg: Thyroglobulin
PTC: Papillary thyroid cancer
WHO: World Health Organization
TSH: Thyroid-stimulating hormone
T4: Thyroxine
TPO Ab: Anti-thyroid peroxidase antibody
TT: Total thyroidectomy
CLND: Central lymph node dissection
RLN: Recurrent laryngeal nerve
ICD-O: International classification of diseases for oncology
Tg Ab: Thyroglobulin antibodies
CARE: CAse REport guidelines
